# Supplementary material for: SARS-CoV-2 within-host population expansion, diversification and adaptation in zoo tigers, lions and hyenas
Source: Nat Commun. 2025 Dec 13;16:11310. doi: 10.1038/s41467-025-66402-7 (PMC12722721; doi:10.1038/s41467-025-66402-7)
Supplement: Supplementary file 1 — Supplementary Information [file 41467_2025_66402_MOESM1_ESM.pdf]

Supplementary Information

Title

SARS-CoV-2 within-host population expansion, diversification and adaptation in zoo tigers, lions and hyenas

Author list

Laura Bashor[1], Emily N. Gallichotte[1], Michelle Galvan[1], Katelyn Erbeck[2], Lara Croft[3,4], Katelyn Stache[3], Mark D. Stenglein[1], James G. Johnson III[3], Kristy Pabilonia[2], and Sue VandeWoude[1*]

Affiliations

[1] Dept. of Microbiology, Immunology and Pathology, Colorado State University, Fort Collins, CO, USA
[2] Colorado State University Veterinary Diagnostic Laboratories, Fort Collins, CO, USA
[3] Denver Zoo Conservation Alliance, Denver, CO, USA
[4] Independent Consultant, Denver, CO, USA
*corresponding author; sue.vandewoude@colostate.edu

Supplementary Tables:

Table S1. Available below.

Available in Supplementary Data file:

Supplementary Data 1. All mutations detected relative to Wuhan reference sequence, including AY20 characteristic mutations and within-host mutations (relative to tiger reference).
Supplementary Data 2. Characteristic AY.20 mutations from outbreak.info.
Supplementary Data 3. MultiQC summary containing coverage and quality metrics output by the nf-core/viralrecon pipeline.
Supplementary Data 4. Variant table output by the nf-core/viralrecon pipeline.
Supplementary Data 5. Population-level nucleotide diversity measures for full SARS-CoV-2 genomes.
Supplementary Data 6. Gene-level nucleotide diversity measures.
Supplementary Data 7. ARTIC version 4 (V4) primer scheme used to generate a pool of overlapping amplicons that cover the entire SARS-CoV-2 genome. Primers were obtained from Integrated DNA Technologies (catalog number 10011442) and the scheme is also available from: https://github.com/artic-network/primer-schemes/.

Supplementary Figures:

Figures S1 and S2. Available below.

Supplementary Information:

GISAID acknowledgements available below.

Table S1. Denver Zoo animal metadata reproduced with permission from Gallichotte et al. (2025)[16].

Species	Group	Animal ID	Sex	Age*
Amur tiger	---	A	F	11
		B	M	11
African lion	pride 1	A	M	6
		B	M	6
		C	M	6
		D	M	6
	pride 2	E	F	6
		F	F	1
		G	M	5
		H	F	9
		I	M	1
		J	F	9
		K	M	2
Spotted hyena	older clan	A	F	22
		B	M	23
	young clan	C	M	7
		D	F	7

*Age at the time of the outbreak (October-November 2021).

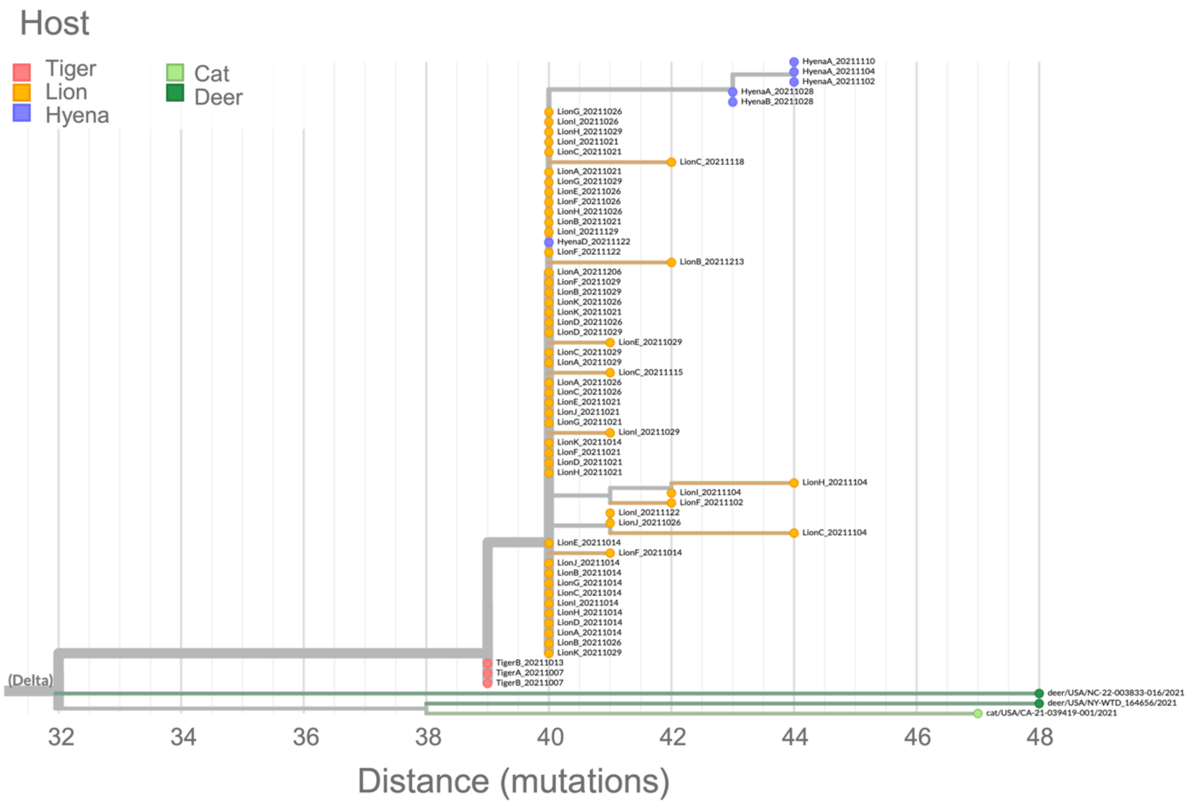

Figure S1. SARS-CoV-2 sequences recovered from Denver Zoo animals are distinct from other sequences from nonhuman animals also classified as AY.20 variants. A time-based phylogenetic tree was generated with Nextstrain and an interactive version is available at https://nextstrain.org/community/laurabashor/DZSARS2. Sequences are colored by host species (Tiger=red, Lion=orange, Hyena=blue, Cat=light green, Deer=dark green) with divergence in number of mutations relative to the ancestral Wuhan-1 reference sequence indicated along the x-axis. Data include SARS-CoV-2 consensus sequences generated from the zoo outbreak (N=63 samples total; N=54 samples from 11 lions, N=3 samples from two tigers and N=6 samples from three hyenas), and all publicly available sequences from animals classified as AY.20 (N=3). Data obtained from GISAID were processed with the Augur pipeline for input into Nextstrain, and acknowledgements are available in EPI_SET_240407tz.

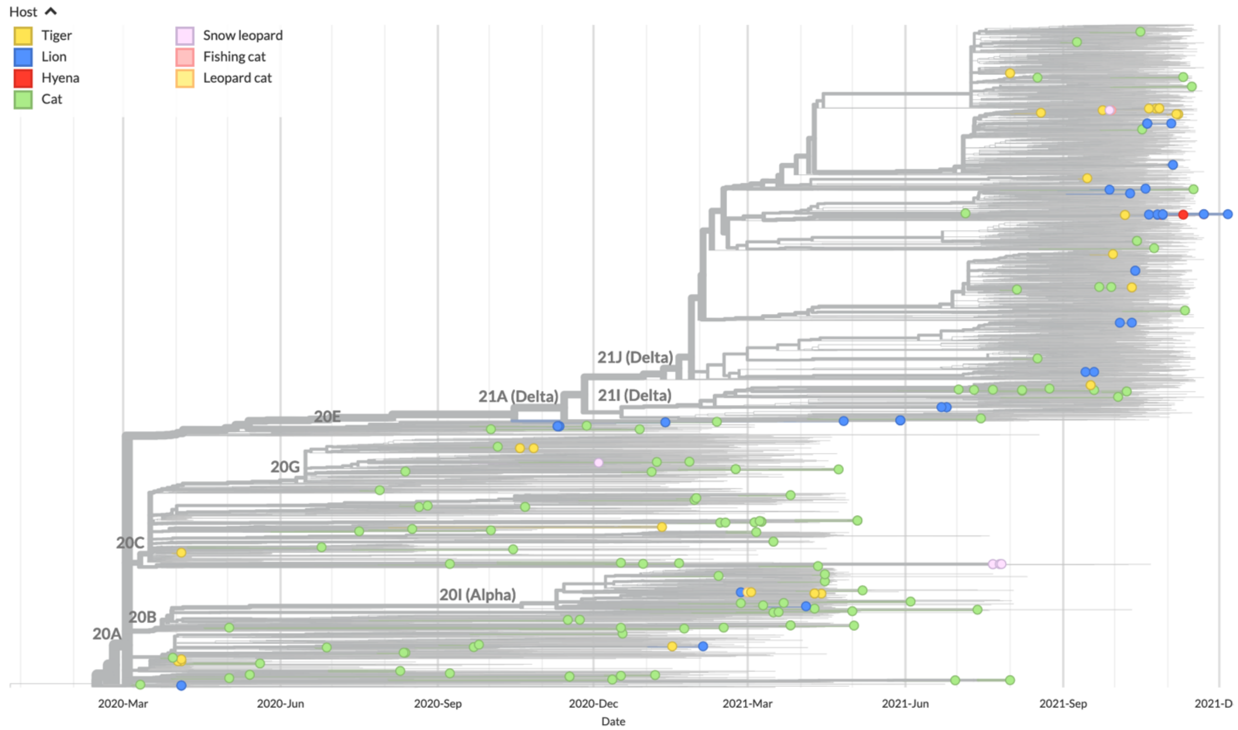

Figure S2. No evidence at the global level for species-specific adaptation of SARS-CoV-2 in felids or hyenas. A time-based phylogenetic tree was generated from all felid- and hyena-derived SARS-CoV-2 sequences available in the GISAID database prior to December 2021, with contextual human-derived sequences collected in the same time and place. Tree was generated with Nextstrain and an interactive version is available at https://nextstrain.org/community/laurabashor/DZSARS2. Sequences are colored by host species and scaled by time, with collection date indicated along the x-axis. The tree included four data inputs: (1) sixteen SARS-CoV-2 consensus sequences from the Denver Zoo outbreak corresponding to the highest quality sequence collected at the latest date from each individual animal (N=16 animals; N=2 tigers, N=11 lions, and N=3 hyenas), (2) a random subsample of human-derived sequences in Colorado from the days leading up to the zoo outbreak (100 sequences/day from September 23rd to October 7th, 2021; N=1500 sequences), (3) all felid-derived SARS-CoV-2 sequences available in the GISAID database prior to December 2021, and (4) contextual sequences collected in the same time and place as each felid sequence (10-100 human-derived sequence per felid-derived sequence) with enriched sampling within the United States. All data obtained from GISAID were processed with the Augur pipeline for input into Nextstrain, and acknowledgements are available in GISAID EPI_SET_240407wv.

Sequences from GISAID included in this study:

We prepared two GISAID EPI_SETs to acknowledge publicly available SARS-CoV-2 sequences included in this study. EPI_SET_240407tz includes all sequences used to generate the phylogenetic tree in Figures 2A, 2B and S1, and additional individual GISAID sequences discussed in the text. EPI_SET_240407wv includes all additional sequences used to generate the phylogenetic tree in Figure S2.

Data Availability
GISAID Identifier: EPI_SET_240407tz
doi: 10.55876/gis8.240407tz
All genome sequences and associated metadata in this dataset are published in GISAID's EpiCoV database. To view the contributors of each individual sequence with details such as accession number, Virus name, Collection date, Originating Lab and Submitting Lab and the list of Authors, visit 10.55876/gis8.240407tz

Data Snapshot
EPI_SET_240407tz is composed of 1,525 individual genome sequences.
The collection dates range from 2019-12-31 to 2021-12-21;
Data were collected in 4 countries and territories;
All sequences in this dataset are compared relative to hCoV-19/Wuhan/WIV04/2019 (WIV04), the official reference sequence employed by GISAID (EPI_ISL_402124). Learn more at https://gisaid.org/WIV04.

Data Availability
GISAID Identifier: EPI_SET_240407wv
doi: 10.55876/gis8.240407wv
All genome sequences and associated metadata in this dataset are published in GISAID's EpiCoV database. To view the contributors of each individual sequence with details such as accession number, Virus name, Collection date, Originating Lab and Submitting Lab and the list of Authors, visit 10.55876/gis8.240407wv

Data Snapshot
EPI_SET_240407wv is composed of 11,999 individual genome sequences.
The collection dates range from 2019-12-31 to 2021-11-22;
Data were collected in 26 countries and territories;
All sequences in this dataset are compared relative to hCoV-19/Wuhan/WIV04/2019 (WIV04), the official reference sequence employed by GISAID (EPI_ISL_402124). Learn more at https://gisaid.org/WIV04.
