# Supplementary material for: SARS-CoV-2 within-host population expansion, diversification and adaptation in zoo tigers, lions and hyenas
Source: Nat Commun. 2025 Dec 13;16:11310. doi: 10.1038/s41467-025-66402-7 (PMC12722721; doi:10.1038/s41467-025-66402-7)
Supplement: Supplementary file 2 — Description of Additional Supplementary Files [file 41467_2025_66402_MOESM2_ESM.pdf]

Description of Additional Supplementary Files

File Name: Supplementary Data 1
Description: All mutations detected relative to Wuhan reference sequence, including AY20 characteristic mutations and within-host mutations (relative to tiger reference).

File Name: Supplementary Data 2
Description: Characteristic AY.20 mutations from outbreak.info.

File Name: Supplementary Data 3
Description: MultiQC summary containing coverage and quality metrics output by the nf-core/viralrecon pipeline.

File Name: Supplementary Data 4
Description: Variant table output by the nf-core/viralrecon pipeline.

File Name: Supplementary Data 5
Description: Population-level nucleotide diversity measures for full SARS-CoV-2 genomes.

File Name: Supplementary Data 6
Description: Gene-level nucleotide diversity measures.

File Name: Supplementary Data 7
Description: ARTIC version 4 (V4) primer scheme used to generate a pool of overlapping amplicons that cover the entire SARS-CoV-2 genome. Primers were obtained from Integrated DNA Technologies (catalog number 10011442) and the scheme is also available from: https://github.com/artic-network/primer-schemes/.
