# Supplementary material for: SARS-CoV-2 within-host population expansion, diversification and adaptation in zoo tigers, lions and hyenas
Source: Nat Commun. 2025 Dec 13;16:11310. doi: 10.1038/s41467-025-66402-7 (PMC12722721; doi:10.1038/s41467-025-66402-7)
Supplement: Supplementary file 4 — Reporting Summary [file 41467_2025_66402_MOESM4_ESM.pdf]

nature portfolio

Corresponding author(s): Sue VandeWoude

Last updated by author(s): Oct 14, 2025

Reporting Summary

Nature Portfolio wishes to improve the reproducibility of the work that we publish. This form provides structure for consistency and transparency in reporting. For further information on Nature Portfolio policies, see our Editorial Policies and the Editorial Policy Checklist.

Statistics

For all statistical analyses, confirm that the following items are present in the figure legend, table legend, main text, or Methods section.

n/a	Confirmed	
☐	☒	The exact sample size (*n*) for each experimental group/condition, given as a discrete number and unit of measurement
☐	☒	A statement on whether measurements were taken from distinct samples or whether the same sample was measured repeatedly
☐	☒	The statistical test(s) used AND whether they are one- or two-sided<br>*Only common tests should be described solely by name; describe more complex techniques in the Methods section.*
☐	☒	A description of all covariates tested
☐	☒	A description of any assumptions or corrections, such as tests of normality and adjustment for multiple comparisons
☐	☒	A full description of the statistical parameters including central tendency (e.g. means) or other basic estimates (e.g. regression coefficient) AND variation (e.g. standard deviation) or associated estimates of uncertainty (e.g. confidence intervals)
☐	☒	For null hypothesis testing, the test statistic (e.g. *F*, *t*, *r*) with confidence intervals, effect sizes, degrees of freedom and *P* value noted<br>*Give P values as exact values whenever suitable.*
☒	☐	For Bayesian analysis, information on the choice of priors and Markov chain Monte Carlo settings
☒	☐	For hierarchical and complex designs, identification of the appropriate level for tests and full reporting of outcomes
☒	☐	Estimates of effect sizes (e.g. Cohen's *d*, Pearson's *r*), indicating how they were calculated

Our web collection on statistics for biologists contains articles on many of the points above.

Software and code

Policy information about availability of computer code

Data collection	No software was used for data collection.
Data analysis	Sequencing data in FASTQ file format were input into the nf-core/viralrecon bioinformatics analysis pipeline version 2.6.0 for detailed quality control metrics, lineage assignment, within-host viral variant calling, variant annotation, and the generation of consensus sequences. The 'tiger reference sequence' used with the the nf-core/viralrecon pipeline was generated with MUSCLE (version 3.8.1551) and emboss (version 6.5.7). This consensus was then used as the reference sequence for running the viralrecon pipeline. A time-based phylogenetic tree was generated with Nextstrain using augur version 13.1.0, auspice version 2.29.1 , and nextstrain-cli version 3.0.3. The tree was visualized in Taxonium version 2.0.227. Within-host virus population demographics and signatures of selection were calculated with SNPGenie version 1.0. All other data visualizations and statistical analyses were carried out in R version 4.3.3 and R Studio version 2023.06.0+421.

For manuscripts utilizing custom algorithms or software that are central to the research but not yet described in published literature, software must be made available to editors and reviewers. We strongly encourage code deposition in a community repository (e.g. GitHub). See the Nature Portfolio guidelines for submitting code & software for further information.

Data

Policy information about availability of data

All manuscripts must include a data availability statement. This statement should provide the following information, where applicable:

- Accession codes, unique identifiers, or web links for publicly available datasets
- A description of any restrictions on data availability
- For clinical datasets or third party data, please ensure that the statement adheres to our policy

All data supporting this study are available in the text and supplementary information. Next-generation sequencing datasets have been deposited in the NCBI Sequence Read Archive and are available under BioProject PRJNA1154601 [https://www.ncbi.nlm.nih.gov/bioproject/?term=PRJNA1154601]. Additional datasheets for analyses included in this study can be found at https://github.com/laurabashor/DZSARS2. Interactive versions and associated metadata for the three phylogenies are available at https://nextstrain.org/community/laurabashor/DZSARS2.

Research involving human participants, their data, or biological material

Policy information about studies with human participants or human data. See also policy information about sex, gender (identity/presentation), and sexual orientation and race, ethnicity and racism.

Reporting on sex and gender	*Use the terms sex (biological attribute) and gender (shaped by social and cultural circumstances) carefully in order to avoid confusing both terms. Indicate if findings apply to only one sex or gender; describe whether sex and gender were considered in study design; whether sex and/or gender was determined based on self-reporting or assigned and methods used. Provide in the source data disaggregated sex and gender data, where this information has been collected, and if consent has been obtained for sharing of individual-level data; provide overall numbers in this Reporting Summary. Please state if this information has not been collected. Report sex- and gender-based analyses where performed, justify reasons for lack of sex- and gender-based analysis.*
Reporting on race, ethnicity, or other socially relevant groupings	*Please specify the socially constructed or socially relevant categorization variable(s) used in your manuscript and explain why they were used. Please note that such variables should not be used as proxies for other socially constructed/relevant variables (for example, race or ethnicity should not be used as a proxy for socioeconomic status). Provide clear definitions of the relevant terms used, how they were provided (by the participants/respondents, the researchers, or third parties), and the method(s) used to classify people into the different categories (e.g. self-report, census or administrative data, social media data, etc.) Please provide details about how you controlled for confounding variables in your analyses.*
Population characteristics	*Describe the covariate-relevant population characteristics of the human research participants (e.g. age, genotypic information, past and current diagnosis and treatment categories). If you filled out the behavioural & social sciences study design questions and have nothing to add here, write "See above."*
Recruitment	*Describe how participants were recruited. Outline any potential self-selection bias or other biases that may be present and how these are likely to impact results.*
Ethics oversight	*Identify the organization(s) that approved the study protocol.*

Note that full information on the approval of the study protocol must also be provided in the manuscript.

Field-specific reporting

Please select the one below that is the best fit for your research. If you are not sure, read the appropriate sections before making your selection.

☒ Life sciences ☐ Behavioural & social sciences ☐ Ecological, evolutionary & environmental sciences

For a reference copy of the document with all sections, see nature.com/documents/nr-reporting-summary-flat.pdf

Life sciences study design

All studies must disclose on these points even when the disclosure is negative.

Sample size	The sample size was predetermined by the number of individuals within each species housed at the zoo (note that the entire population of each species was included in sampling). The longitudinal sampling protocol providing repeated screening and defined the period that viral nucleic acid sequences could be detected as reported in Gallichotte et al. 2025 (https://doi.org/10.1128/msphere.00989-24). We did not carry out a power calculation before collecting samples or to plan an experimental design because samples were collected for surveillance purposes, and all animals within an exhibit were sampled.
Data exclusions	Replicate NGS datasets that had greater than or equal to 75% 1X and 10X coverage and less than 25,000 Ns per 100kb were retained for within-host variant analysis. This led to the exclusion of three datasets generated from blank RNA extraction negative control samples, 16 datasets corresponding to both technical replicates for eight biological samples, and two single replicate datasets corresponding to two other biological samples (Lion I on November 22nd and Lion C on November 4th).
Replication	Biological replicates were limited by the number of animals of each species housed at the zoo. Technical sequencing replicates were used for

	quality control (see data exclusions) and measuring within-host variation.
Randomization	Not relevant as this was an observational study.
Blinding	Not relevant as this was an observational study.

Reporting for specific materials, systems and methods

We require information from authors about some types of materials, experimental systems and methods used in many studies. Here, indicate whether each material, system or method listed is relevant to your study. If you are not sure if a list item applies to your research, read the appropriate section before selecting a response.

Materials & experimental systems

n/a	Involved in the study
☒	☐ Antibodies
☒	☐ Eukaryotic cell lines
☒	☐ Palaeontology and archaeology
☐	☒ Animals and other organisms
☒	☐ Clinical data
☒	☐ Dual use research of concern
☒	☐ Plants

Methods

n/a	Involved in the study
☒	☐ ChIP-seq
☒	☐ Flow cytometry
☒	☐ MRI-based neuroimaging

Animals and other research organisms

Policy information about studies involving animals; ARRIVE guidelines recommended for reporting animal research, and Sex and Gender in Research

Laboratory animals	The study did not involve laboratory animals.
Wild animals	The study involved African lions, Amur tigers, and Spotted hyenas living at Denver Zoo. Animal age is reported in Table S1.
Reporting on sex	Animal sex is reported in Table S1.
Field-collected samples	This study did not involve field-collected samples.
Ethics oversight	This research was approved by Denver Zoo Conservation Alliance (DZCA) through the Association of Zoos & Aquariums standardized research proposal process (internal DZCA approval number 2023-11).

Note that full information on the approval of the study protocol must also be provided in the manuscript.

Plants

Seed stocks	N/A
Novel plant genotypes	N/A
Authentication	N/A
